# Druggability and Binding Site Interaction Studies of Potential Metabolites Isolated from Marine Sponge Aurora globostellata against Human Epidermal Growth Factor Receptor-2

**DOI:** 10.6026/97320630013261

**Published:** 2017-08-31

**Authors:** M. Sugappriya, D. Sudarsanam, Raj Bhaskaran, Jerrine Joseph, Arumugam Suresh

**Affiliations:** 1 Research and development centre, Bharathiar University, Coimbatore 641 046, Tamilnadu, India; 2 Department of Zoology and Advanced Biotechnology, Loyola college, Chennai 600034,Tamil nadu, India; 3 School of Biotechnology and genetic engineering Bharathiar University, Coimbatore 641 046, Tamilnadu, India; 4 Centre for Drug Discovery and Development, Jeppiaar Research park, Sathyabama University, Chennai 600119,Tamilnadu, India

**Keywords:** Docking, ADME, HER2, XP, SP, Aurora globostellata

## Abstract

To study the involvement of compounds stigmasterol and oleic acid isolated from marine sponge Aurora globostellata and docking against the Human Epidermal Growth Factor Receptor-2 in breast cancer. The comparative molecular docking was performed with the natural compounds from marine sponge and the synthetic drugs used in breast cancer treatment against the target HER2. The molecular docking analysis was done using GLIDE in Schrodinger software package. The ADME properties were calculated using the Qikprop. The observation of the common binding site for all the ligands confirms the binding pocket; where the isolated compound Stigmasterol agrees well with the binding residues and thus can be optimized further to arrive at a molecule that has a high binding affinity and low binding constant. The results of the docking studies carried out on HER2 provide an insight for the compound stigmasterol to have drug like properties than oleic acid. These results are supportive to confirm the marine sponges as a better lead for cancer therapeutics.

## Background

The ocean is the elixir of life. Its composition is an excellent resource to be tapped for drug discovery. The marine environment is complex with variations in pressure, salinity, temperature and biological habitats. The marine organisms have unique therapeutic properties. These have been explored and are yet to be proved [1]. Approximately one half of the total global biodiversity is represented by marine organisms, which are the reservoirs of active natural products [2]. The organisms living in oceans are unique with richest sources of new drug leads. Marine sponges are said to be the gold mines for the past 50 years, with respect to the diversity of secondary metabolites. Sponges produce wide array of compounds with varying carbon skeleton, by which the diseases can be suppressed at different points on focusing specific targets. The secondary metabolites produced are biologically active molecules not directly involved in normal functions of the organisms, which includes growth, reproduction or development [2, 3].

The pharmaceutical interest in sponges arouse in the early 1950s with the discovery of spongothymidine and spongouridine nucleosides from marine sponge cryptotethia crypta [4] These were the basis for the synthesis of Ara-C which is the first marine derived anticancer compound and Ara- A the antiviral drug [5, 6]. Ara-c is used for the treatment of leukaemia and lymphoma, the derivatives of Ara-C is used for various cancer types. It has been found that the lipid components such as fatty acids, sterols and other unsaponifiable compounds occur in lower invertebrates than higher animals.

In olden days, sponges were soaked with wine and put on the left side for heartaches, and sponges soaked in urine are used for the treatment of bites of poisonous animals. In 18th century, the physicians used sponges in powdered form for lung diseases, which comprise of various types of sponges mixed together and powdered. The sponge Spongia officinalis is used as syrup for dry and asthmatic cough in western parts of the world. Manoalide, the sesterterpenoids isolated from marine sponge Luffariella variabilis [7], is found to be an antibiotic and analgesic. There are around 5300 different products discovered from sponges. The ability to stimulate the production of secondary metabolites by sponges is an important consideration when one wants to harvest compounds from sponges for the production of potential novel therapeutics. The molecular mode of action is not thoroughly investigated, whereas the mechanism by which the sponges interfere with compounds have been reported [8], through which the bioactive compounds can be transformed into new medicines.

Here, in this study, the marine sponge Aurora globostellata is considered based on its importance in pharmaceutical applications (manuscript communicated). The compounds isolated have been characterized in detail for breast cancer. Their bioactivity is explored in in-vitro and in-vivo studies. The attempt has been undertaken to evaluate the mode of action and druggability of the metabolites isolated and characterized. The discovery of number of bioactive compounds from sponges has been increasing day by day. The natural source would overcome the existing synthetic drugs in mode of action and also reduce the side effects caused by the commercial compounds. Based on the 3D structure of the receptors, modern methods of discovering new leads from natural source are on the rise. The present study focuses on the in-silico analysis of the naturally isolated compounds from marine sponges and compared with the results for the commercial drugs: Afinitor, Halaven, Ixabipilone, Lapatinib, Letrozole, Palbocilib, Raloxifene, and Tamoxifen. The in-silico approach enables one to screen for ADMET properties of vast number of molecules within a few minutes thus reducing the time and is a non- expensive and non-tedious process with great accuracy, which is not possible in standard experimental methods [9, 10, 11]. A comparative analysis of the compounds using Glide Schrodinger package is used to find the common binding residues in HER2, the breast cancer target from among the ten considered compounds. This can confirm the quality of the natural compounds with high binding affinity than the commercial drugs [12, 13].

The marine sponges are collected from Rameswaram Coast, Tamil Nadu by SCUBA diving and they are extracted using hexane solvent. The compounds are isolated using column chromatography and the identification of the isolated compounds is accomplished using spectroscopic methods like GCMS and NMR. The compounds are confirmed as Stigmasterol and Oleic acid; these two compounds are considered to be the ligands for docking analysis against Human Epidermal Growth Factor Receptor 2 (RCSB PDB code 1N8Z). A comparison of the docking results of the breast cancer drugs with the natural compounds isolated from marine sponges Aurora globostellata, against the HER2 has been carried out to estimate the quality of the isolated compounds to act as drug like molecules equivalent to that of the commercial drugs.

## Methodology

### Preparation of target

HER-2 / neu have been widely studied in breast cancer. The HER-2/ neu oncogene encodes a transmembrane tyrosine kinase receptor with extensive homology to the epidermal growth factor receptor 2. HER2 receptors consists of four transmembrane tyrosine receptors, they are HER1, also called as ErbB1, HER2 (ErbB2), HER3 (ErbB3) and HER4 (ErbB4) [14]. HER2 is a gene responsible for breast cancer, it is also called as ERBB2 (Erb-B2 receptor tyrosine kinase). The over expression of HER2 protein makes the uncontrollable growth and division of cancer cells. The HER2 is found to be over expressed in 20-25% cases. The ErbB receptors contains four plasma membrane receptor tyrosine kinase and all these members of the family contain extra cellular domains, the dimerization site and the ligand binding site where the synthetic molecule binds [15, 16].

The protein three-dimensional crystal structure of Human Epidermal Growth Factor Receptor 2 (PDB ID 1N8Z) is obtained from Protein Data bank and is prepared for the analysis, using protein preparation wizard. In the protein preparation step, protein minimization, grid generation and docking of ligands were done using Glide Schrodinger package [11]. The Hydrogen atoms were added to the protein for maintaining the ionization and tautomeric state of Asp, Glu, Ser, His and Arg amino acids. The missing side chains and atoms are corrected, followed by the protein structure minimization using force fields to minimize the steric clashes in the structure. This protein structure was used for the grid generation in further docking analysis.

### Preparation of Ligand

The commercial compounds Afinitor, Ixabipilone, Letrozole, Halaven, Lapatinib, Palbociclib, Raloxifene and Tamoxifen and the natural compounds isolated from Aurora globostellata, Stigmasterol and Oleic acid are considered as the ligands against the target HER-2. The ligand structures are downloaded from Pubchem. The ligands have been segregated into three groups; the first group consisting of the five commercial compounds Afinitor, Ixabipilone, Letrozole, Halaven, Lapatinib; the second group representing the next three potential commercial compounds (Palbociclib, Raloxifene and Tamoxifen) and the last group as the isolated compounds (Stigmasterol and Oleic acid). Ligprep was used for ligand preparation. It generates various structures with ionization states at pH 7.0±2.0 with ionizer. The force field Merck Molecular Force field MMFF94 is used for the optimization, producing low energy conformation of the ligand [18].

### Maestro

The package Maestro from SchrÖdinger used here has various merits, where it supports various file formats as structural input, featured tool in creating molecular models and has shown to possess a high visualization capability in viewing small to large complex molecules [19].

### Glide (Grid based Ligand Docking with Energetics):

Glide focuses towards the orientation of the molecule, its position and the conformation, which screen large libraries. Glide docking applies three different scoring functions; they are Standard precision docking (SP), High throughput virtual screening (HTVS) and Extra precision docking (XP). Both HTVS and SP docking use the same scoring function. The HTVS minimize the immediate conformations throughout docking, and reduces the torsional refinement and more suitable for screening more ligands. XP docking is found to be superior to SP docking in terms of sampling. XP docking reduces the false positive and has more additional terms than SP. In the docking methodology, initially Glide uses hierarchical filters for finding the active site regions for ligand binding in the receptor molecules. Poses means the alignment, position and conformation with respect to the receptor. The next step is the ligand screening, which is an exhaustive search based on torsion angle space. After the ligand screening, it is minimized using molecular mechanics energy function, which is said to be a reasonable model in prediction of binding modes [20]. The best poses are given by E-model score which deals with the van der Waals and electrostatic forces. Glide score represents the buried polar groups and steric clashes, which ranks different ligand poses, where the more negative value represents the tighter binding affinity [13].

### ADME Profiling

The lead compounds from natural resources fail to enter into the market due to the poor pharmacokinetic properties. So, designing ligands satisfying the Adsorption, Distribution, Metabolism and Elimination (ADME) properties will go through the market as a good drug. The drugs should be orally absorbed and distributed to the site of action and eliminated from the body without leaving any traces, which produces adverse effects. Hence, the tools and computer-aided methods, nowadays, have become popular in identifying good drug candidate molecule [21, 22, 23].

### ADMET related descriptors

QikProp, the package in Schrodinger is used for calculating molecular descriptors in predicting ADMET properties [24]. The following parameters are considered here with their ranges given specifically; Polar Surface Area (PSA) that is related to oral bioavailability with the area less than 140A2; Rule of Five indicating the molecules suitability for oral administration; QPlog BB- Blood Brain Barrier that provides an access for the central nervous system with a range lies between -3.0 to 1.0; QPlogPo/w that calculates the hydrophobicity of the molecule with a range of 2.0-6.5; QPlogHERG, the experimental IC50 value for HERG K+ channel blockage, with a range below -5.0; QPPCaco and QPPMDCK, the respective cell permeabilities with a value of >500 nm/sec [25].

## Results

### Docking Analysis

Molecular docking approach helps us in identifying best binding ligands with the protein target and helps in exploring new small molecular leads from natural sources with higher binding affinities. These lead molecules enter into the higher phases of drug development and may end up as a good drug candidate. The protein ligand interactions were carried out using Schrodinger (GLIDE) commercial software. The target protein, the crystal structure of extracellular domain of human HER2, complexed with Herceptin Fab, was considered for this analysis. The Herceptin Fab domain was removed for the docking of commercial drugs with HER2. The ligands considered are: the commercial drugs, Afinitor, Halaven, Ixabipilone, Lapatinib, Letrozole, Palbociclib, Raloxifene and Tamoxifen and the natural compounds Oleic acid and Stigmasterol isolated from marine sponge Aurora globostellata. In this study, XP Docking procedure was used. It ranks the best conformations based on the ligand binding to the receptor molecules.

### Comparison of Ligands

The docking results of the ten ligands including the natural compounds have been listed in Table 1. The Gscore is a scoring function that predicts the binding energy of the ligand; it ranks the different poses of the ligands. The higher the negative score shows the higher and tight binding affinity. From this study, the compounds are ranked as follows based on their binding energies: Afinitor > Ixabipilone > Letrozole > Halaven > Palbocilib > Oleic acid > Raloxifene > Lapatinib > Stigmasterol > Tamoxifen. From the comparison of the docking energetics, it is observed that the Gscore values are all in the same range, indicating that they all can be grouped into a single family. Except Afinitor, Halavan, Ixabipilone and Letrozole, all others form a cluster to be like a drug. This indicates that the natural compounds, Oleic Acid and Stigmasterol behave like a drug like molecules. The same grouping is confirmed from the point of view of van der Waals, electrostatics, internal, hydrogen bonding and binding energies as well. Similar residues seem to have hydrogen bonding, indicate the closeness in the grouping.

### Analysis of Ligand Druggability

**Figure 1:**
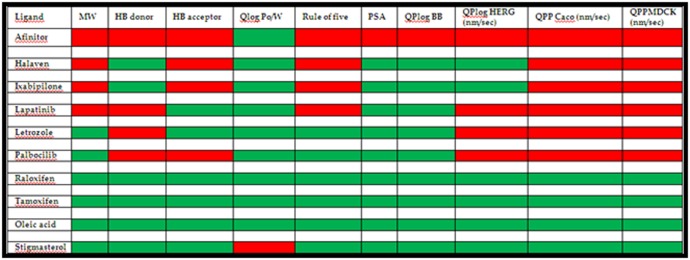
Tabular Diagram indicating the ligands that are successfully having the drug-like and ADME properties (Green block).

As per Lipinski's rule, the parameters for the drug-like property for the ligands have been listed and compared Figure 1. The first four commercial drugs have a higher molecular weight by not obeying the rule. Afinitor, Lapatinib, Letrozole and Palbociclib show higher donor HB and hence do not obey the rule. The first four and Palbociclib show a negative red band indicating their non-drug like behavior. Surprisingly, except Stigmasterol all the others show a positive drug-like property of QlogPo/w. In the Overall sense, Rule of Five shows a non-drug like nature for the first four commercial drugs. PSA is negative for Afinitor. Likewise, QPlog BB shows the same trend. The ADME properties, Qplog HERG, QPP Caco and QPPMDCK show a nondrug like nature for all the ligands except Raloxifene, Tamoxifen, Stigmasterol and Oleic acid. Thus, the comparison of the ligands based on the Lipinski's rule and ADME properties indicate a strong drug likeness for the best commercial drugs Raloxifene and Tamoxifen that have been observed with the green blocks for all the properties in the tabular diagram, Figure 1. Coincidently, the isolated compounds, Stigmasterol and Oleic acid show the same nature as these two commercial drugs and expected to behave in the same manner as the drug like molecules. This would be evaluated by the in-silico method by the interaction studies with the target, as discussed in the following section.

### Docking Interactions and Binding Site Analysis

**Figure 2:**
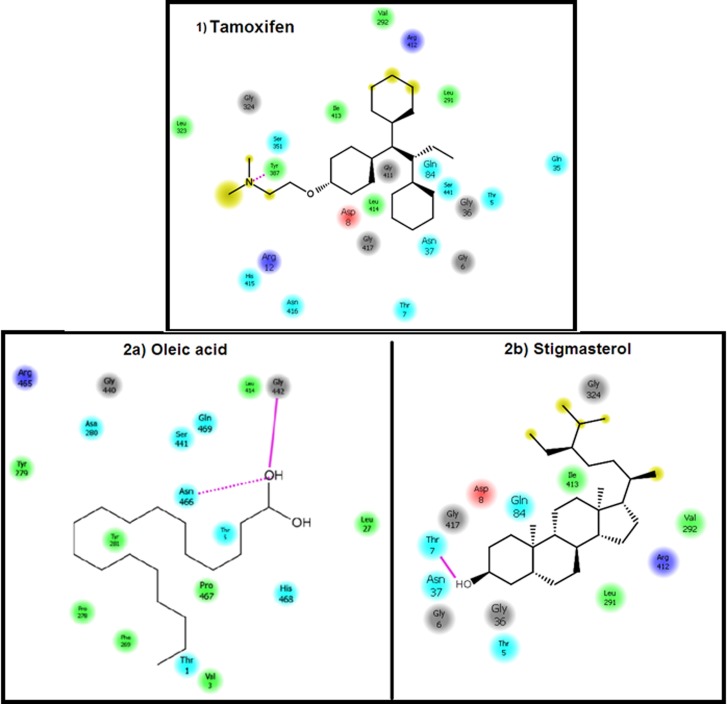
Interaction maps of the ligands with HER2. 1) Commercial drug Tamoxifen; 2) Isolated compounds Oleic acid (2a) and Stigmasterol (2b).

The docking interactions map between HER2 with all the ten ligands provides the information about the interacting residues and their mode and type of interactions with the ligands under consideration. The interaction maps are shown only for three ligands, namely Tamoxifen, Oleic acid and Stigmasterol shown in Figure 2.

## Discussion

**Figure 3:**
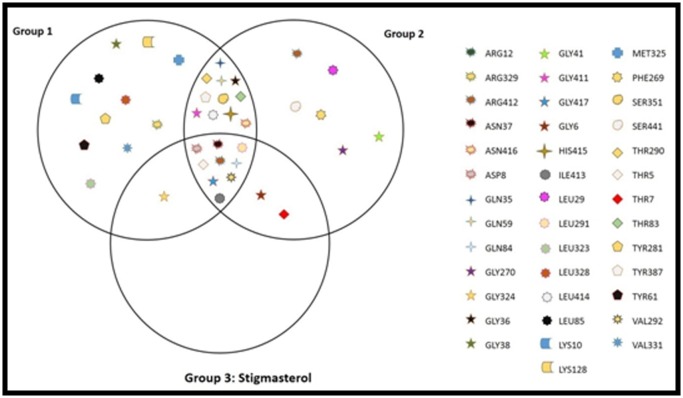
Common Ligand Binding Sites

The residues of HER2 that have closer interactions with the ligands are highlighted in different colored circles based on the type of interactions such as hydrogen bonds, Van der Waals forces, ionic bonds. These were the residues responsible for the ligand-HER2 interactions respectively. All these interacting residues for all the ligands are identified and the common interacting residues are obtained to figure out the pharmacophore / functional interacting pattern. To obtain this, the ligands are grouped into three categories: the first five of the commercial drugs, the next three - the most potent commercial drugs and lastly, the isolated compounds from Sponge, namely Stigmasterol and Oleic acid. For all the three groups, their respective binding site residues are identified. Then a Venn diagram has been drawn, which is as shown in Figure 3.

The first group contains 32 residues, the second groups have 28 residues and the third groups have 12 residues in common among the ligands in the groups. The Venn diagram shows 9 residues as common among all the groups / ligands and 23 residues as common among any two of the three groups. The isolated compounds contain almost all of their interacting residues with HER2. The following 9 residues are common among all the ligands: Thr5, Asp8, Asn37, Gln84, Leu291, Val292, Arg412, Ile413 and Gly417.

**Figure 4:**
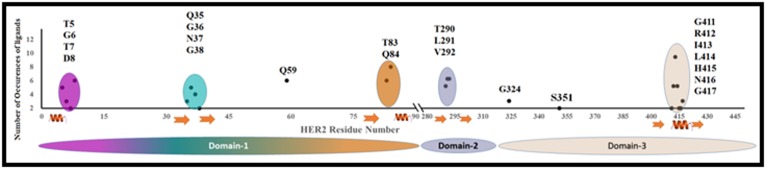
Sequential Distribution of Binding Site Residues.

We plotted a scatter diagram (Figure 4) of the cumulative occurrences of these ligands interacting with the residues of HER2 (plotted against the sequence number of HER2). The resultant plot clearly indicates that there are five different clusters in HER2 that are so closer to one among them spatially, in the folded form, thus forming the pocket of interaction. The clusters are colored differently so as to differentiate them on the structure. These clusters are distributed among the three domains: Nterminal Domain, middle elongated domain and the third helical domain. The N-terminal domain consists of first three of the clusters (residues T5, G6, T7 and D8; residues Q35, G36, N37 and G38; residues T83 and Q84); middle domain has the cluster 4 (residues T290, L291 and V292), and the helical domain 3 has the helical cluster (residues G411, R412, I413, L414, H415, N416 and G417). It has been observed that the domain 1 consists majorly of polar residues and domain 2 is observed with hydrophobic residues. The helical domain 3 has both hydrophobic and hydrophilic residues and the helical wheel plot segregates both the groups for specific interactions.

**Figure 5:**
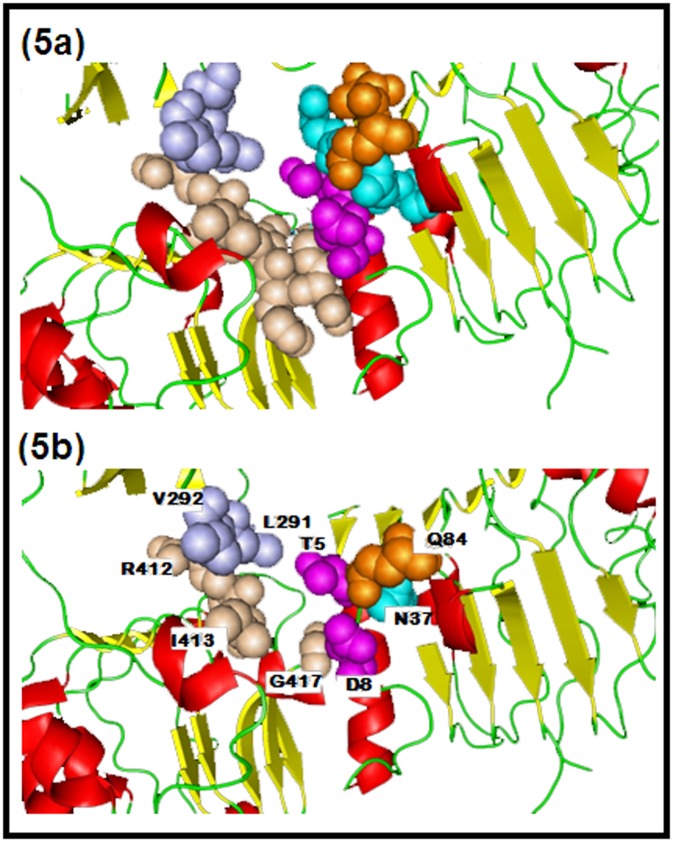
HER2 binding site residues: 5a) Arrangement of 23 common residues; 5b) Nine common binding site.

When these 23 residues that are common among either two of the groups are highlighted on the structure of HER2, it clearly shows (Figure 5a), the converged region / binding pocket on the structure wherein these ligands bind with HER2. The cluster group residues are colored the same way as in Figure 3. When the pocket has been filled with only the 9 common residues (Figure 5b), it clearly indicates the functional groups that form the pocket to which the drug is expected to bind. The designing of a drug is based on these functional groups and is to be optimized so as to get a high affinity molecule with lowest binding constant.

The observation of the pocket of interaction that is common among the ligands confirms the binding pocket, which when optimized to design a molecule that fits well within the pocket forms the initiation of the design of a candidate molecule. The isolated compound Stigmasterol agrees well with the binding residues and thus can be optimized further to arrive at a molecule that has a high binding affinity and low binding constant.

## Conclusion

Thus, the results of the docking studies carried out on HER2 corroborate to the findings that the most suitable drug like properties are possessed by Stigmasterol. In comparison with Oleic acid it is a better bet as oleic acid is more lipophilic commonly present in sponges, which is relatively less druggable. This provides evidence of how a marine sponge can be a source of potential anti- cancer agent. Further in-vivo studies need to be performed in future to validate the wet lab results. The preclinical studies will pave way for a potential anti-cancer compound.

## Figures and Tables

**Table 1:** Comparison of Ligand Docking Energetics.

Ligand Energetics (in kCal / Mole)	XP Gscore	Glide Evdw	Glide Ecoul	Glide Einternal	XP Hbond	dG Bind	Residues forming Hydrogen Bonds
Afinitor	-8.907	-45.777	-14.285	7.405	-5.356	-70.476	Val 331, Leu 85, Gln 59
Halaven	-5.563	-36.89	-5.053	0	-1.753	-61.14	Val 331, Tyr 387,Gln 84
Ixabipilone	-6.331	-29.125	-10.046	4.061	-2.872	-63.783	Gln 59, Gln 84
Lapatinib	-4.616	-39.745	-13.474	5.805	-2.88	-92.722	Gly 270, Gln 59, Gln 84
Letrozole	-5.629	-31.567	-7.523	7.358	-1.509	-57.2	Gly 270
Palbociclib	-4.842	-30.651	-11.181	15.147	-1.575	-77.059	Gly 270, Asp 8
Raloxifene	-4.652	-46.522	-6.133	10.766	-0.732	-65.82	Leu 414, Arg 12
Tamoxifen	-3.983	-32.468	-4.258	6.151	-0.7	-77.837	Tyr 387
Oleic acid	-4.747	-26.798	-5.938	8.468	-3.252	-76.227	Gly 442, Asn 466
Stigmasterol	-4.291	-27.255	-4.698	2.017	-0.785	-71.338	Thr 7
